# Supplementary material for: Oligodendrocyte precursor cells facilitate neuronal lysosome release
Source: Nat Commun. 2025 Jan 30;16:1175. doi: 10.1038/s41467-025-56484-8 (PMC11782495; doi:10.1038/s41467-025-56484-8)
Supplement: Supplementary file 1 — Supplementary Information [file 41467_2025_56484_MOESM1_ESM.pdf]

Supplementary information for

Oligodendrocyte precursor cells facilitate neuronal lysosome release

Li-Pao Fang[1,2,3], Ching-Hsin Lin[3,4#], Yasser Medlej[5#], Renping Zhao[6], Hsin-Fang Chang[3,4], Qilin Guo[1], Zhonghao Wu[7], Yixun Su[7,8], Na Zhao[1,9], Davide Gobbo[1], Amanda Wyatt[3,10], Vanessa Wahl[3,10], Frederic Fiore[11], Szu-Min Tu[3,4], Ulrich Boehm[3,10], Wenhui Huang[1], Shan Bian[12], Amit Agarwal[11], Marcel A. Lauterbach[5], Chenju Yi[8], Jianqin Niu[7], Anja Scheller[1,3], Frank Kirchhoff[1,3*], Xianshu Bai[1,2,3*]

1. Molecular Physiology, Center for Integrative Physiology and Molecular Medicine (CIPMM), University of Saarland, 66421 Homburg, Germany
2. State Key Laboratory of Natural Medicines, Department of Pharmacology, School of Pharmacy, China Pharmaceutical University, Nanjing 211198, China
3. Center for Gender-specific Biology and Medicine (CGBM), University of Saarland, 66421 Homburg, Germany
4. Cellular Neurophysiology, CIPMM, University of Saarland, 66421 Homburg, Germany
5. Molecular Imaging, CIPMM, University of Saarland, 66421 Homburg, Germany
6. Biophysics, CIPMM, University of Saarland, 66421 Homburg, Germany
7. Research Centre, Seventh Affiliated Hospital of Sun Yat-sen University; Shenzhen, 518107, China
8. Department of Histology and Embryology, Chongqing Key Laboratory of Neurobiology, Brain and Intelligence Research Key Laboratory of Chongqing Education Commission, Third Military Medical University, Chongqing 400038, China
9. Present address: Institute of Anatomy and Cell Biology, University of Saarland, 66421 Homburg, Germany
10. Experimental Pharmacology, Center for Molecular Signaling (PZMS), Saarland University School of Medicine, Homburg, Germany
11. The Chica and Heinz Schaller Research Group, Institute for Anatomy and Cell Biology, Heidelberg University, Heidelberg, Germany
12. Institute for Regenerative Medicine, State Key Laboratory of Cardiology and Medical Innovation Center, Shanghai East Hospital, Frontier Science Center for Stem Cell Research, School of Life Sciences and Technology, Tongji University, Shanghai, China.

# These authors contributed equally to the work.

* : Correspondence should be addressed to

Prof. Frank Kirchhoff: frank.kirchhoff@uks.eu,

Dr. Xianshu Bai xianshu.bai@uks.eu

Supplementary Methods

Animals

NG2-CreER x CAG-LSL-hM3Dq-pta-mCitrine x Rosa26-LSL-tdTomato[1-3] mice were maintained in the animal facility of the institute for Anatomy and Cell Biology, Heidelberg University, Germany. Tamoxifen in sunflower seed oil (100mg/kg/day) was administered for 5 consecutive days at the age of 4 weeks[3] (**Suppl. Fig. 5**).

TgH (NG2-CreER^{T2})[4] mice carrying Rosa26-^{fl}STOP^{fl}-GCaMP3 (NG2$^{CT2\text{-}GCaMP3}$)[5] or CAG-^{fl}CTA^{fl}-EGFP reporter (NG2$^{CT2\text{-}EGFP}$)[6] were maintained in C57Bl/6N background and used for morphological analysis (**Suppl. Fig. 3; Suppl. Fig. 4J**). Tamoxifen in Miglyol®812 (100mg/kg bodyweight, Caesar & Lorentz GmbH, Hilden, Germany) was administered to NG2$^{CT2\text{-}GCaMP3}$ mice at the age of 5 weeks (w) for five consecutive days and analyzed at 9 w, while for NG2$^{CT2\text{-}EGFP}$ mice was administered at the age of 11 w for one day and analyzed four days later .

AAV injection

Adult mice at the age between 8-12 weeks were administered with 10 mg/kg of Carprofen one hour before the surgery. Mouse was fixed on a stereotactic apparatus under continuous inhalational isoflurane (5% for induction and 2% for maintenance with mixture of O_2 and N_2O), and the eyes were covered by Bepanthen (Bayer). After sterile cleaning and skin incision, for intracortical injection, the skull was then thinned laterally by 1.5 mm and longitudinally by 1.8-2 mm from Bregma using a dental drill. AAV8-hSynapsin1-hM3D(Gq)-mCherry (Addgene, #50474, ⩾ 2 x 10^{12} vg/ml) in a volume of 0.3 µl was injected intra-cortically with a rate of 0.1 µl/min at a depth of 0.6 mm from pia. The syringe was kept in place for 5 min after the injection to avoid liquid reflux. Following the surgeries, the incision was sutured and analgesia was administered intraperitoneally daily for three consecutive days for postoperative pain management. Animals injected with virus or KA also received Tramal (0.4 mg/ml) in the drinking water for the first seven days or Buprenorphin (1 mg/kg) *ad libitum* in the drinking water for the first three days after the injection, respectively.

CNO administration *in vivo*

After three-four weeks of successful microinjection of AAV8-hSynapsin1-hM3D(Gq)-mCherry, mice were administered with intraperitoneal injection of CNO (5 mg/kg in saline, helloBio) for five consecutive days. Mice were sacrificed 2 hours after the last CNO injection.

For NG2-CreER x CAG-LSL-hM3Dq-pta-mCitrine x Rosa26-LSL-tdTomato mice, two weeks after the last tamoxifen injection, mice were administered CNO intraperitoneally (1mg/kg in saline, HelloBio) for five consecutive days and were sacrificed 2 days after the last CNO injection[3].

Calcium imaging and analysis in primary neurons

To indicate neuronal activity, genetically encoded calcium indicator was delivered to the neurons using AAV-hSyn-GCaMP6m-XC (Addgene, #118975). At 1 DIV, 2ml of AAVs (≥2 × 10^{11} vg/ml) was added to each well of the 6-well plate. At 7 DIV, AAV8-hSynapsin1-hM3D(Gq)-mCherry (≥2 × 10^{11} vg/ml) was added to each well (2 ml/well) for manipulation of neuronal activity combined with CNO.

Neurons at 12-14 DIV were placed in the imaging chamber of LSM 780 confocal microscope with consistent temperature (37 °C) and CO_2 supply (5%). Live imaging was performed with 40× objective at single layer with a recording frequency of 4.16 Hz. The baseline was recorded for 4 minutes, followed by another 4 minutes recording after CNO (40 μM) application.

Calcium recording from neuronal cultures were analyzed using Fiji ImageJ. Whole-field fluorescence signal trace (Z-axis profile) was processed using the ImageJ plug-in Find Peaks after drifting correction and using the auto-threshold background calculation. Peak amplitude (calculated as $\Delta F/F0$) and frequency before and after CNO application were compared using a two-tailed paired t-test.

Supplementary Figures

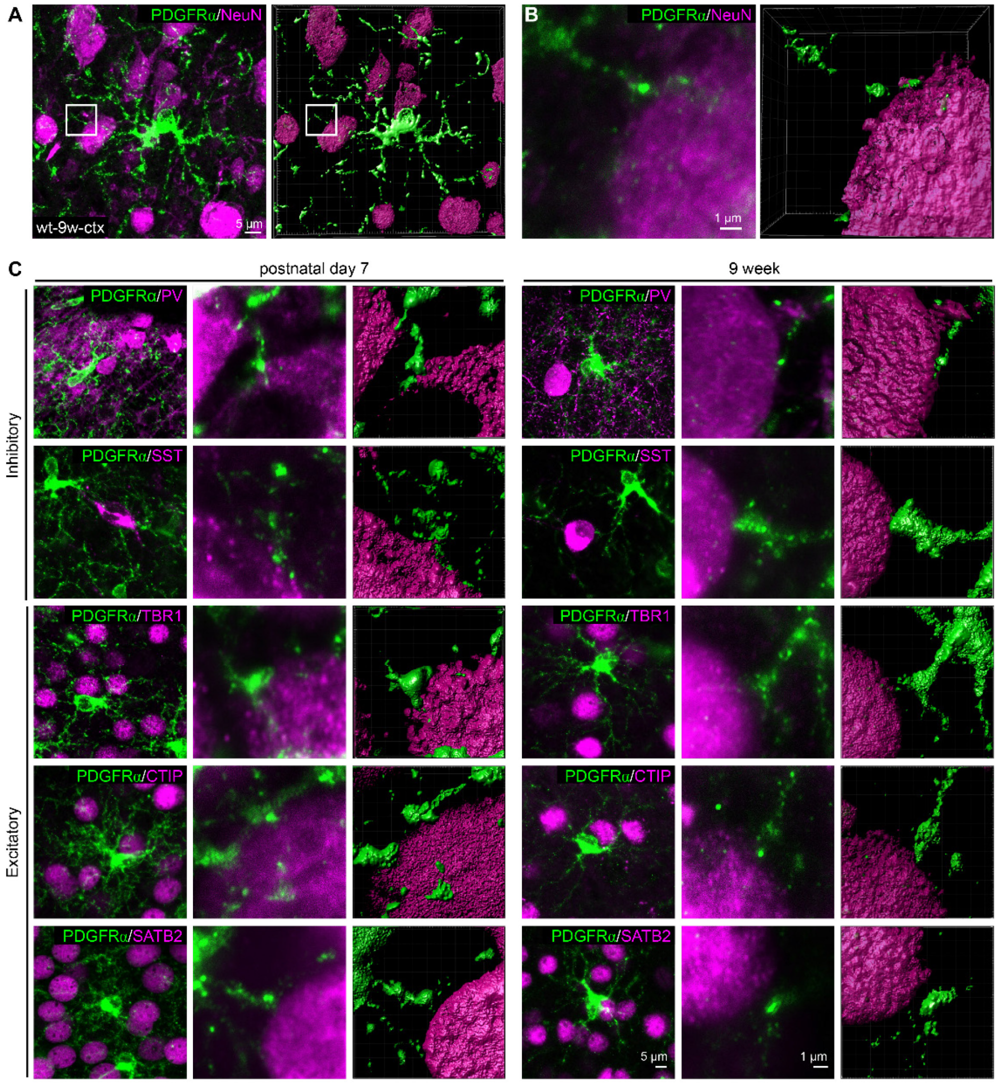

Supplementary Figure 1. OPCs form proximal junction with neurons in the developing and adult cortex.

A, B Immunostaining of OPCs and neurons with PDGFRα and NeuN in the cortex of adult (9-week) wild type mouse. **C** Immunostaining of OPCs and various types of neurons combining PDGFRα with Parvalbumin (PV), somatostatin (SST), T-box brain transcription factor 1 (TBR1), COUP-TF interacting protein (CTIP), Special AT-rich sequence-binding protein 2 (SATB2) in the developing (postnatal day 7) and adult brain.

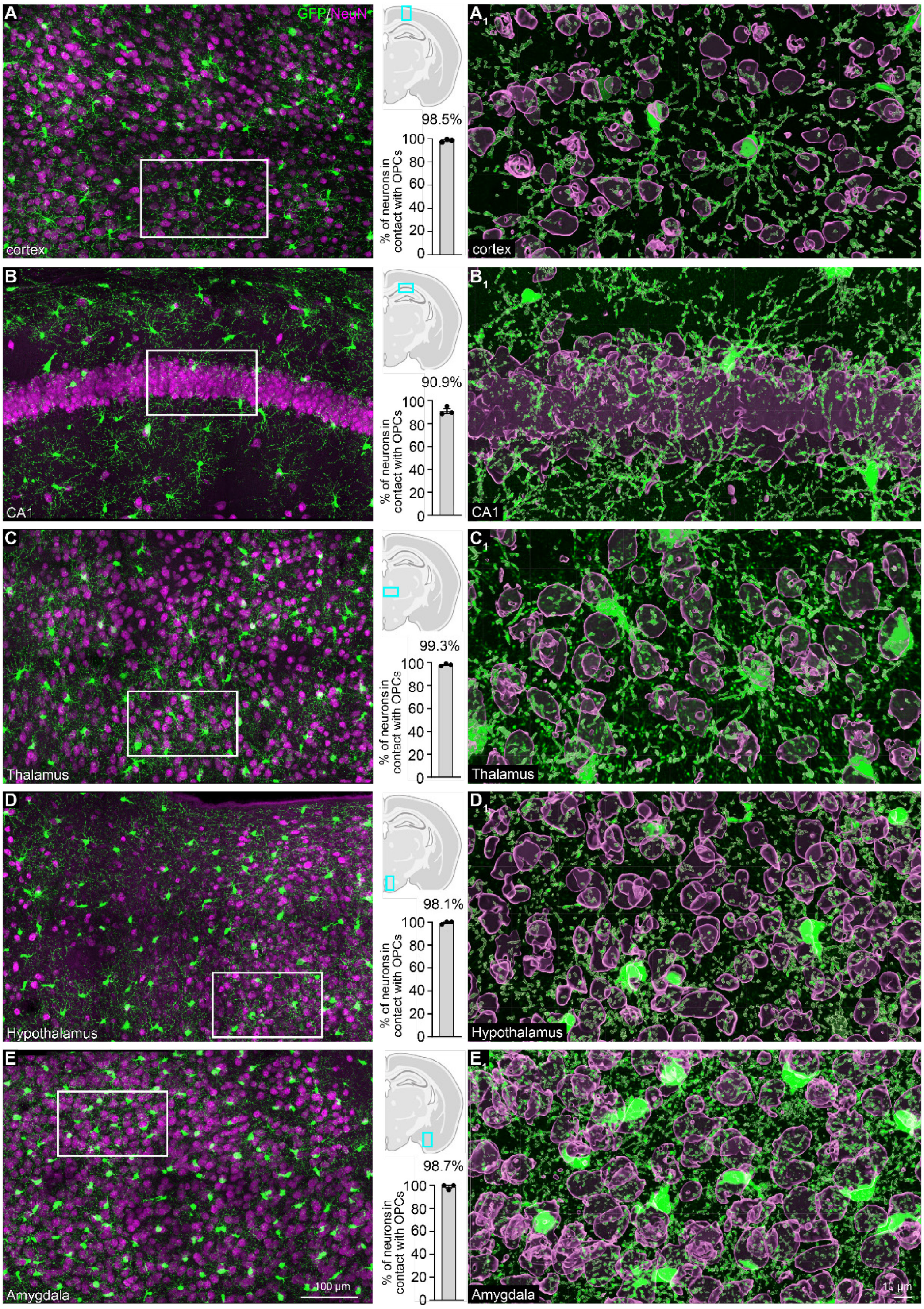

Supplementary Figure 2. OPC-neuron junction is a common feature in all regions of grey matter.

A-E Immunostaining (left panel) and 3D-recostruction of OPCs and neurons with GFP and NeuN antibodies from cortex (**A**), hippocampal CA1 region (**B**), thalamus (**C**), hypothalamus (**D**) and amygdala (**E**) of NG2-EYFP mice. Middle panels show the percentage of neurons

receiving OPC contact in corresponding region. Data are shown as mean ± SEM. Source data are provided as a Source Data file.

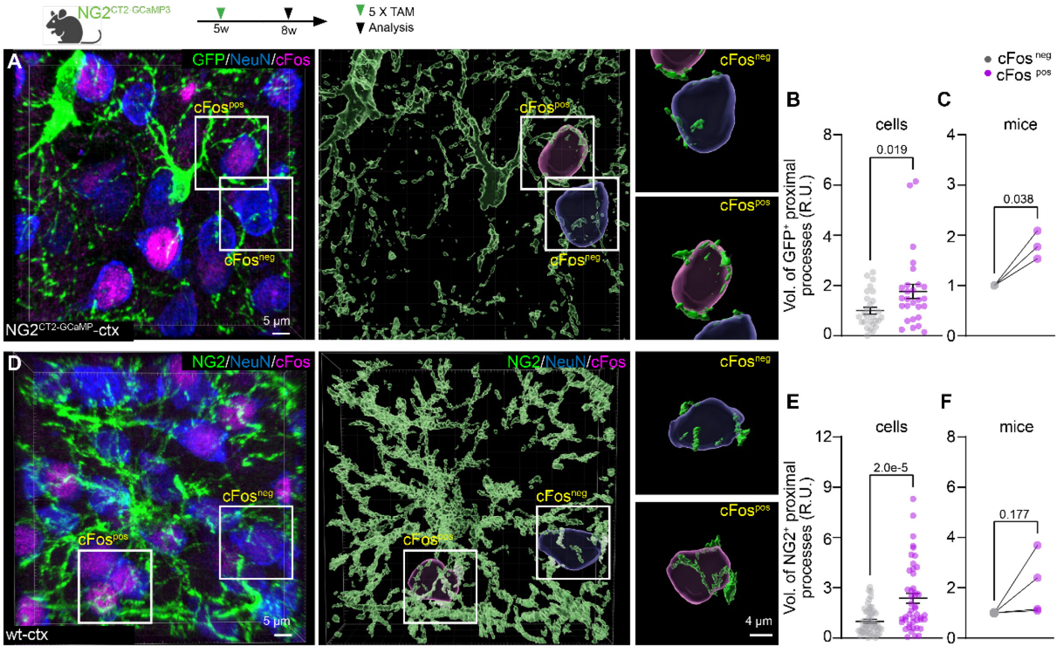

Supplementary Figure 3. OPCs preferentially form contact with active neurons.

A, D Immunostaining of OPCs with GFP (**A**) or NG2 (**D**), along with markers of cellular activity (cFos) and neurons with NeuN antibodies in the cortex of NG2-CreER^{T2} x GCaMP3 (NG2^{GCaMP3}) mice (**A**) or wild type mice (**D**), respectively. **B, C, E, F** Quantification of relative volume of OPC processes (GFP^{+}) in contact with cFos^{pos} and cFos^{neg} neurons. (**B:** cFos^{neg} =27 cells, cFos^{pos}=27 cells, two-tailed unpaired t-test; **C**: cFos^{neg}=3 mice, cFos^{pos}=3 mice, two-tailed paired t-test; **E**: cFos^{neg}=50 cells, cFos^{pos}=48 cells, two-tailed unpaired t-test; **F**: cFos^{neg}=4 mice, cFos^{pos}=4 mice, two-tailed paired t-test). R.U.=relative unit, normalized to control. Data are shown as mean ± SEM. Source data are provided as a Source Data file. Created in BioRender. Fang, L. (2025) https://BioRender.com/f32o880

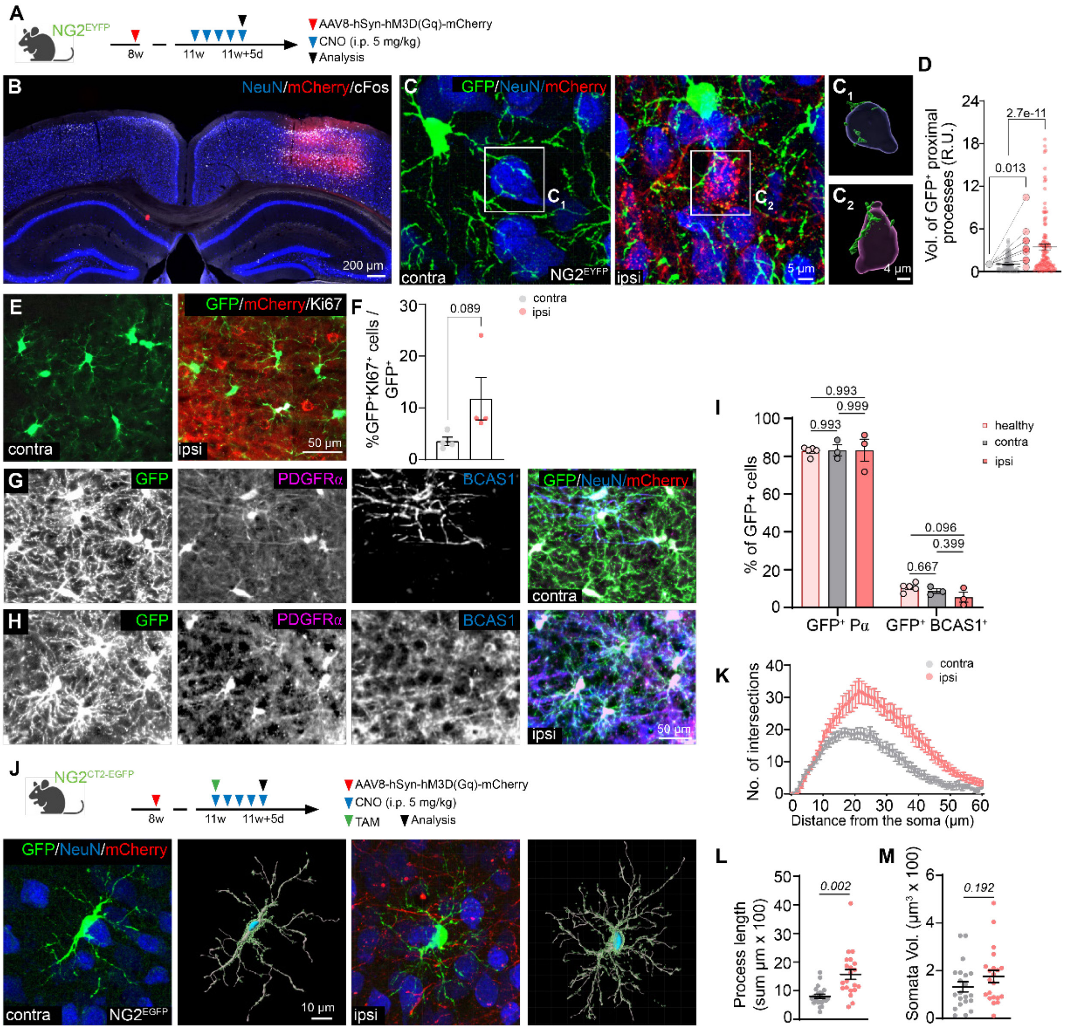

Supplementary Figure 4. Chemogenetic activation of neurons triggers OPC-neuron contact formation.

A Scheme of experimental plan. **B** Overview of coronal brain slices from NG2^{EYFP} mice, intracortically injected with AAV8-hSyn-hM3D(Gq)-mCherry virus, immunostained for NeuN and cFos. **C** Immunostaining and 3D-reconstruction of OPCs and neurons with GFP and NeuN antibodies in contra- and ipsilateral cortex. **D** Analysis of the volume of OPC processes in contact with mCherry^{+} and mCherry^{-} neuronal somata (contralateral: 154 cells from 5 mice, ipsilateral: 111 cells from 5 mice, two-tailed unpaired t-test for cells and two-tailed paired t-test for mice). R.U.=relative unit, normalized to control. **E** Immunostaining of proliferating OPCs with GFP and Ki67 at the contra- and ipsilateral cortex of virus injected NG2^{EYFP} mouse. **F** Percentage of proliferating OPCs (GFP^{+}Ki67^{+}) among total GFP^{+} cells. **G** Immunostaining of OPCs and immature oligodendrocytes (OLs) with PDGFRα (magenta), BCAS1 (blue) and GFP (green) in the contra- and ipsilateral cortex of NG2^{EYFP} mice after three weeks of intracortical injection of AAV8-hSyn-hM3D(Gq)-mCherry. **I** Percentage of GFP^{+} OPCs (GFP^{+}PDGFRα^{+}) and GFP^{+} immature OLs (GFP^{+}BCAS1^{+}) in the healthy, contra- and

ipsilateral cortex of $NG2^{EYFP}$ mice (healthy: (OPC: 82.7 ± 1.0%; immature OL: 10.6 ± 1.0%; N=5 mice); contralateral: (OPC: 83.1 ± 2.8%; immature OL: 8.7 ± 1.2%; N=3 mice); ipsilateral: (OPC: 83.2 ± 5.9%; immature OL: 5.4 ± 2.6%; N=3 mice; One-way ANOVA). **J-M** Immunostaining and 3D-reconstruction of single OPC with GFP antibody in contra- and ipsilateral side of NG2-$CreER^{T2}$ x CAG-^{fl}CTA^{fl}-EGFP mice ($NG2^{CT2\text{-}EGFP}$). **K-M** Morphological analysis of OPCs in contra- and ipsilateral cortex, including the number of intersections (**K**), total length of the processes (**L**) and the volume of OPC-somata (**M**) (contra-=21 cells and ipsi-=21 cells from 4 mice; two-sided unpaired t-tests). Data are shown as mean ± SEM. Source data are provided as a Source Data file. Created in BioRender. Fang, L. (2025) https://BioRender.com/f32o880

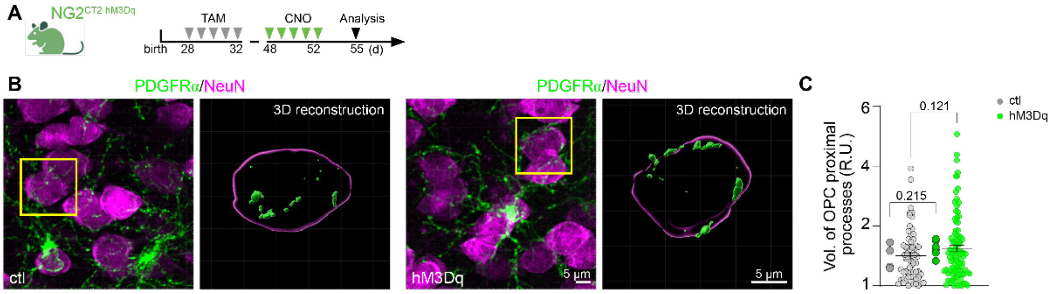

Supplementary Figure 5. Chemogenetic activation of OPCs does not change the contact frequency between neuronal somata and OPC processes.

A Scheme of the experiment. **B** Immunostaining and 3D-reconstruction of neurons (NeuN^{+}) and OPCs (PDGFRα^{+}) in the control and NG2-CreER x CAG-LSL-hM3Dq-pta-mCitrine (NG2$^{CT2\text{-}hM3Dq}$) mice. **C** Analysis of the relative volume of OPC processes in contact with neuronal somata (ctl=66 cells from 4 mice, hM3Dq=97 cells from 4 mice, two-tailed unpaired t-test). Data are shown as mean ± SEM. Source data are provided as a Source Data file. Created in BioRender. Fang, L. (2025) https://BioRender.com/f32o880

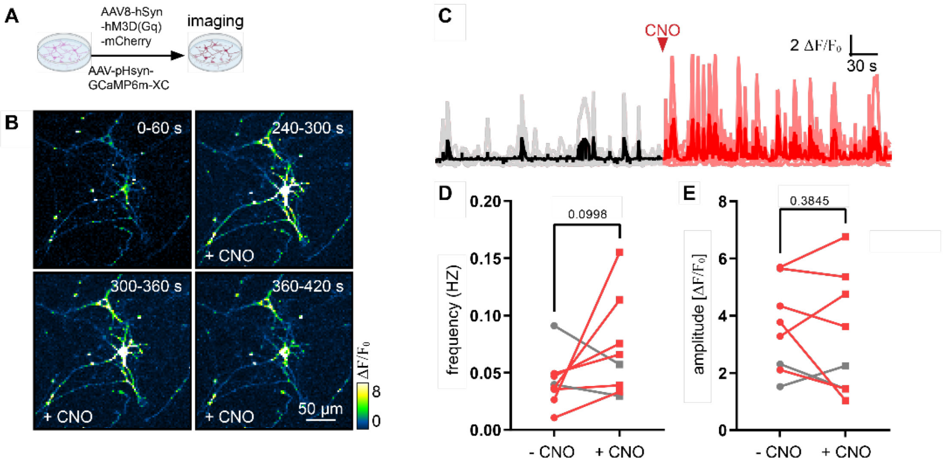

Supplementary Figure 6. Chemogenetic activation triggers calcium activity in primary neurons.

A Scheme of the experiment. **B** Exemplary microimages of primary neurons transfected with calcium indicator with AAV-pHsyn-GCaMP6m-XC. Cells were also transfected with AAV8-hSyn-hM3D(Gq)-mCherry. Calcium imaging was performed before and after Clozapine-N-oxide (CNO) application. **C** Traces of neuronal calcium events before (black line for mean of 8 traces, grey line for each single trace) and after CNO application (dark red line for mean of 8 traces, light red line for each single trace). **D, E** Quantitative analysis of the frequency and amplitude of neuronal calcium activity before and after CNO application (n=8 cells from 3 independent experiments, one mouse/experiment; two-tailed paired t-test). About 75% of cells responded to CNO (6 cells out of 8). Source data are provided as a Source Data file. Created in BioRender. Fang, L. (2025) https://BioRender.com/f32o880

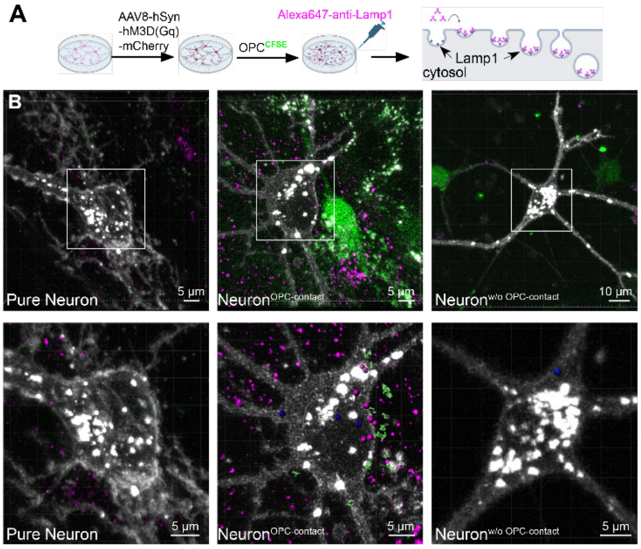

Supplementary Figure 7. OPC contact facilitates neuronal lysosome release. (Original images of Fig. 2G).

A Scheme of the experiment. **B** Live-imaging of lysosome exocytosis in neurons from pure neuronal culture and neurons with (neuron$^{\text{OPC-contact}}$) or without OPC contact (neurons$^{\text{w/o-OPC-contact}}$) from co-culture. In neurons$^{\text{OPC-contact}}$, the Alexa647^{+} puncta were classified into proximal (<1 µm, magenta) and distal (>1 µm, blue) based on their distance to OPC contact site. Created in BioRender. Fang, L. (2025) https://BioRender.com/f32o880

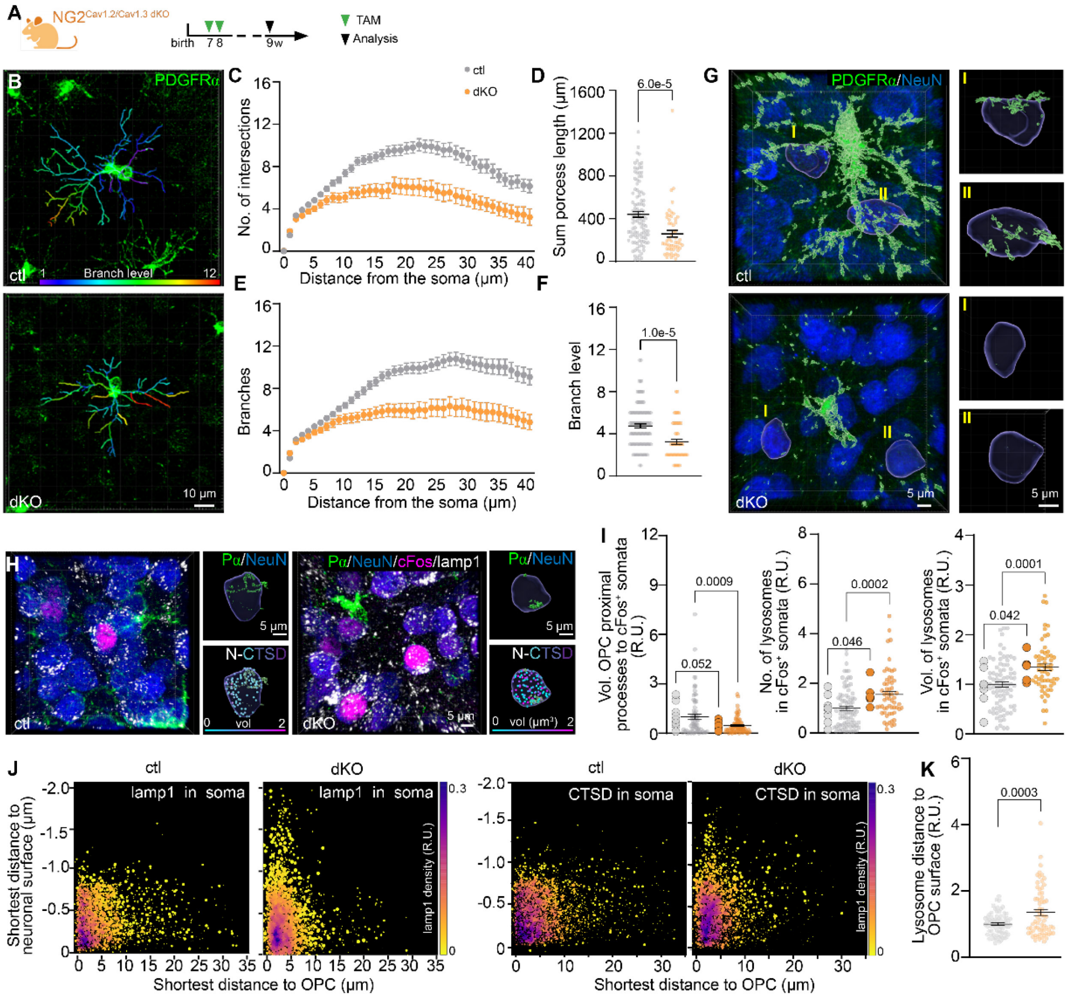

Supplementary Figure 8. Reduced contacts between OPC processes and neuronal somata correlates with neuronal lysosome accumulation.

A Experimental scheme for double conditional deletion of Cav1.2 and Cav1.3 in OPCs (dKO). **B** Exemplary images of OPCs stained with PDGFRα and 3D-reconstructed. **C-F** Morphological analysis of OPCs from control (ctl) and dKO mouse cortex, including the mean number of intersections on concentric circles every 1 μm from OPC somata (**C**), total length of OPC processes (**D**), the highest level of branches on concentric circles every 1 μm from OPC somata (**E**), and mean branch levels of OPCs (**F**). (**C-F**: ctl=108 cells from 4 mice, dKO=54 cells from 3 mice, two-tailed unpaired t-test). **G** Immunostaining and 3D-reconstruction of OPCs and neurons with PDGFRα and NeuN antibodies in ctl and dKO mice cortex. **H** Immunostaining and 3D-reconstruction of OPCs and neurons with PDGFRα and NeuN in ctl and dKO mice cortex. Neuronal activity was indicated with cFos immunoreactivity (magenta). **I** Analysis of the relative volume of OPC processes in contact with cFos^{+} neurons in ctl and dKO mouse cortex (ctl=74 cells from 9 mice, dKO=74 cells from 8 mice, two-tailed unpaired t-tests). Comparison of the relative number and mean volume of lysosomes in cFos^{+}

neurons between ctl and dKO mice (ctl=84 cells from 9 mice, dKO=59 cells from 6 mice, two-tailed unpaired t-tests). **J** Plot of lysosomal volume and shortest distance to OPC surface and neuronal surface. Lysosomes were identified based on Lamp1 or Cathepsin D (CTSD) immunoreactivity. The relative volume of each lysosome is indicated by the size of the circle and the density of the lysosomes at each position were indicated by the colour code, with the purple indicating higher density (lamp1: ctl=1313 lysosomes from 4 mice, dKO=1100 cells from 4 mice, CTSD: ctl=1321 lysosomes from 4 mice, dKO=1077 cells from 4 mice, K: ctl=73 lysosomes from 4 mice, dKO=68 cells from 4 mice, two-tailed paired t-test). **K** Quantitative analysis of shortest distance between lysosomes and OPC surface. Data are shown as mean ± SEM. Source data are provided as a Source Data file. Created in BioRender. Fang, L. (2025) https://BioRender.com/f32o880

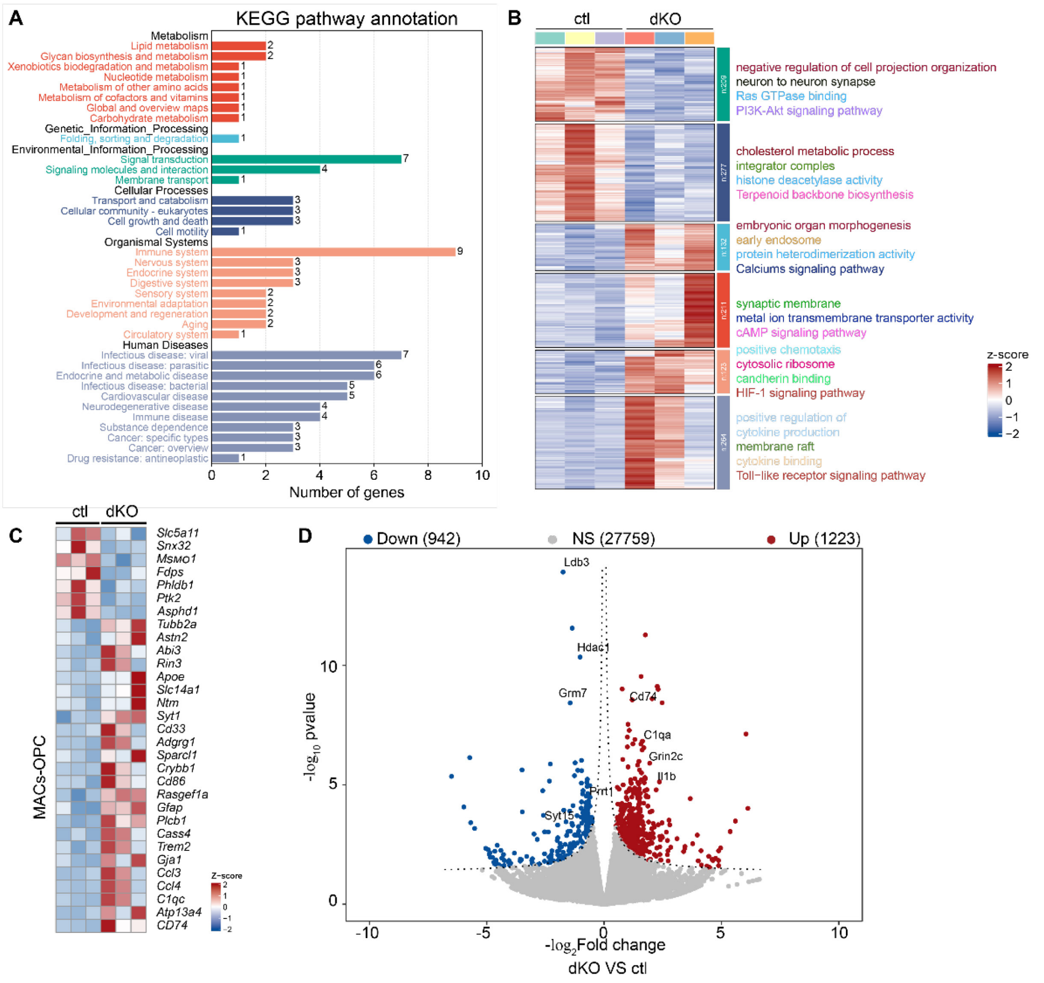

Supplementary Figure 9. Transcriptomic analysis of cortical OPCs from Cav1.2/Cav1.3 double knockout mice.

A KEGG pathway enrichment of differentially expressed genes (DEGs) in OPCs of dKO mouse cortex compared to control. **B** Heatmap of all the DEGs from the OPCs of control and dKO mouse cortex, which were classified into 6 clusters. **C** Comparison of Alzheimer's disease-related genes differentially expressed in OPCs from ctl and dKO mouse cortex. **D** Volcano plot of DEGs identified between control and dKO OPCs. Red dots indicate DEGs with more than 1 fold increase (eg. *Cd74*: padj=1.96E-6; *C1qa*: padj=1.67E-4; *Grin2c*: padj=2.610E-4; *Il1b*: padj=7.44E-4); Blue dots indicate DEGs with more than 1 fold decrease (eg. *Ldb3*: padj=1.12E-10; *Hdac*: padj=1.62E-7; *Prrt1*: padj=0.01; *Syt15*: padj=0.012); Grey dots indicates unchanged DEGs. Source data are provided as a Source Data file.

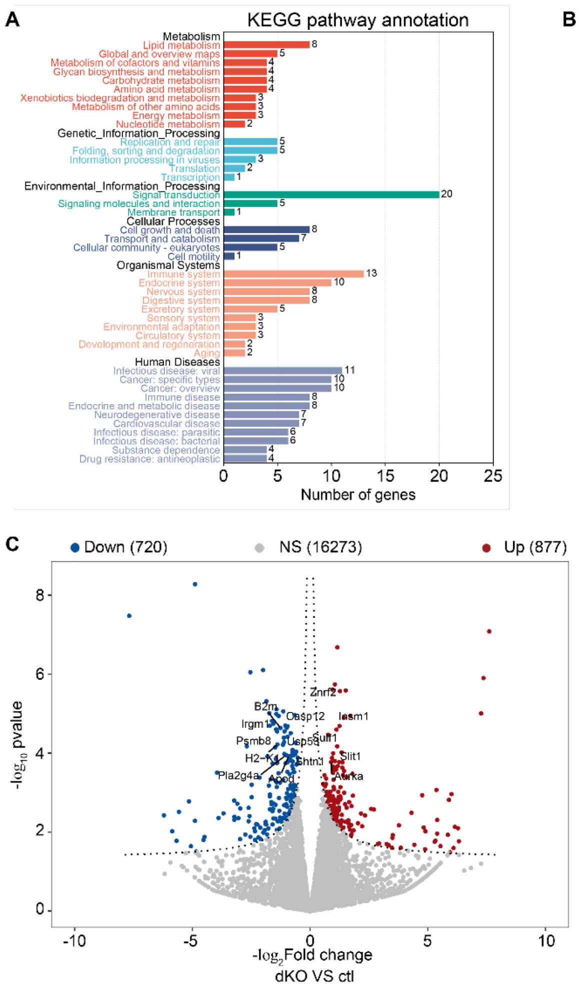

Supplementary Figure 10. Transcriptomic analysis of neurons from Cav1.2/Cav1.3 double knockout mouse cortex.

A KEGG pathway enrichment of differentially expressed genes (DEGs) in neurons of dKO mouse cortex compared to control. **B** Heatmap of all the DEGs from the neurons of control and dKO mouse cortex, which were classified into 5 clusters. **C** Red dots indicate DEGs with more than 1 fold increase (eg. *Znrf2*: padj=4.36E-3; *Insm1*: padj=0.0138; *Sulf1*: padj=0.015; *Slit1*: padj=0.028; *Aurka*: padj=0.044); Blue dots indicate DEGs with more than 1 fold decrease (eg. *B2m*: padj=0.014; *Irgm1*: padj=0.015; *Casp12*: padj=0.015; *Psmb8*: padj=0.015; *H2-K1*: padj=0.027; *Usp53*: padj=0.033; *Pla2g4a*: padj=0.036; *Apod*: padj=0.037); Grey dots indicates unchanged DEGs. Source data are provided as a Source Data file.

Supplementary Tables

Supplementary Table 1. List of primary antibodies.

Antibodies	Host	Dilutions	Cat. No.	Company
**Cathepsin D**	Rabbit	1:500	ab75852	abcam
**cFos**	Guinea pig	1:1000	226004	Synaptic Systems
**CTIP**	Rat	1:500	650601	Biolegend
**GABA**	Rabbit	1:500	20094	Immunostar
**GAD67**	Mouse	1:500	MAB5406	Millipore
**GFP**	Goat	1:1000	600-101-215	Rockland
**GFP**	Rabbit	1:1000	632593	Clontec
**IBA1**	Rabbit	1:1000	019-19741	Wako
**IBA1**	Goat	1:500	ab5076	abcam
**Lamp1**	Rat	1:250	121602	Biolegend
**NeuN**	Rabbit	1:500	ab104225	abcam
**NeuN**	Mouse	1:250	MAB377	Millipore
**NG2**	Rat	1:100		Trotter Lab
**p16INK4a**	Mouse	1:250	MA5-17142	Invitrogen
**Parvalbumin**	Mouse	1:500	P3088	Sigma
**PDGFRα**	Goat	1:500	AF1062	R&D Systems
**SATB2**	Rabbit	1:500	ab92446	abcam
**Somatostatin**	Rat	1:250	MAB354	Millipore
**TBR1**	Rabbit	1:500	49661	Cell Signaling

Supplementary Table 2. List of secondary antibodies.

Antibodies	Fluorophore	Dilutions	Cat. No.	Company
Donkey anti-mouse	Alexa Fluor® 488	1:1000	A21202	Thermo Fisher
	Alexa Fluor® 546	1:1000	A10036	
	Alexa Fluor® 647	1:1000	A31571	
	DyLight® 755	1:1000	SA5-10171	Invitrogen
Donkey anti-rabbit	Alexa Fluor® Plus 405	1:1000	A48258	Thermo Fisher
	Alexa Fluor® 488	1:1000	A21206	
	Alexa Fluor® 546	1:1000	A10040	
	Alexa Fluor® 647	1:1000	A31573	
	Alexa Fluor® 790	1:1000	A11374	
Donkey anti-goat	Alexa Fluor® 488	1:1000	A11055	Thermo Fisher
	Alexa Fluor® 546	1:1000	A11056	
	Alexa Fluor® 647	1:1000	A21447	
	Alexa Fluor® 750	1:1000	ab175744	abcam
Donkey anti-rat	Alexa Fluor™ Plus 405	1:250	A48268	Invitrogen
	DyLight-755	1:500	SA5-10031	Thermo Fisher
Donkey anti- guinea pig	Alexa Fluor® 488	1:500	706-545-148	Jackson Immuno Research Europe Ltd
	Alexa Fluor® 647	1:500	706-605-148	
Goat anti-rat	ATTO® 647N	1:250	40839	Sigma-Aldrich
Goat anti- rabbit	ATTO® 647N	1:100	612-156-120	Rockland

1 Zhu, X. *et al.* Age-dependent fate and lineage restriction of single NG2 cells. *Development* **138**, 745-753 (2011). https://doi.org/10.1242/dev.047951

2 Zhu, H. *et al.* Cre-dependent DREADD (Designer Receptors Exclusively Activated by Designer Drugs) mice. *Genesis* **54**, 439-446 (2016). https://doi.org/10.1002/dvg.22949

3 Fiore, F. *et al.* Norepinephrine regulates calcium signals and fate of oligodendrocyte precursor cells in the mouse cerebral cortex. *Nat Commun* **14**, 8122 (2023). https://doi.org/10.1038/s41467-023-43920-w

4 Huang, W. *et al.* Novel NG2-CreERT2 knock-in mice demonstrate heterogeneous differentiation potential of NG2 glia during development. *Glia* **62**, 896-913 (2014). https://doi.org/10.1002/glia.22648

5 Paukert, M. *et al.* Norepinephrine controls astroglial responsiveness to local circuit activity. *Neuron* **82**, 1263-1270 (2014). https://doi.org/10.1016/j.neuron.2014.04.038

6 Nakamura, T., Colbert, M. C. & Robbins, J. Neural crest cells retain multipotential characteristics in the developing valves and label the cardiac conduction system. *Circ Res* **98**, 1547-1554 (2006). https://doi.org/10.1161/01.RES.0000227505.19472.69
