# Supplementary material for: Oligodendrocyte precursor cells facilitate neuronal lysosome release
Source: Nat Commun. 2025 Jan 30;16:1175. doi: 10.1038/s41467-025-56484-8 (PMC11782495; doi:10.1038/s41467-025-56484-8)
Supplement: Supplementary file 2 — Description of Additional Supplementary Files [file 41467_2025_56484_MOESM2_ESM.docx]

**Description of Additional Supplementary Files**

**Supplementary Video 1 - 2:** Live imaging was performed to monitor OPC process recruitment towards neuronal somata before (**1**) and after (**2**) chemogenetic stimulation of neurons. Neurons (white) were transfected with AAV8-hSyn-h3MD(Gq)-mCherry at 7 days *in vitro* (DIV), while OPCs labeled with CellTrace-FarRed (magenta) were introduced at 12 DIV. At 14 DIV, cells were imaged for one hour to establish a baseline, followed by an additional hour of imaging after the application of 40 µM Clozapine-N-oxide (CNO) to the medium.

**Supplementary Video 3:** Live imaging was conducted to observe neuronal lysosome trafficking near the OPC contact site. OPCs were labeled with CellTrace-FarRed (magenta), and lysosomes were visualized using LysoTracker (green). Neurons were transfected with AAV8-hSyn-h3MD(Gq)-mCherry (red) for enhanced visualization. Cells were imaged using confocal laser scanning microscopy (LSM) for 30 minutes.

**Supplementary Video 4 - 6:** Live imaging was performed to observe lysosome exocytosis in neurons under three conditions: in neuronal culture alone (**4**), neurons in contact with OPCs in co-culture (**5**), and neurons without OPC contact in co-culture (**6**). Neurons were transfected with AAV8-hSyn-h3MD(Gq)-mCherry (white), while OPCs were labeled with CellTrace-CFSE (green). To visualize lysosomal exocytosis, Alexa647-conjugated anti-LAMP1 antibody (magenta) was introduced into the medium, binding to the luminal domain of LAMP1, which becomes exposed during exocytosis.
