# Supplementary material for: Oligodendrocyte precursor cells facilitate neuronal lysosome release
Source: Nat Commun. 2025 Jan 30;16:1175. doi: 10.1038/s41467-025-56484-8 (PMC11782495; doi:10.1038/s41467-025-56484-8)
Supplement: Supplementary file 9 — Reporting Summary [file 41467_2025_56484_MOESM9_ESM.pdf]

nature portfolio

Corresponding author(s): Frank Kirchhoff, Xianshu Bai

Last updated by author(s): Jan 7, 2025

Reporting Summary

Nature Portfolio wishes to improve the reproducibility of the work that we publish. This form provides structure for consistency and transparency in reporting. For further information on Nature Portfolio policies, see our Editorial Policies and the Editorial Policy Checklist.

Statistics

For all statistical analyses, confirm that the following items are present in the figure legend, table legend, main text, or Methods section.

n/a	Confirmed	
☐	☒	The exact sample size (*n*) for each experimental group/condition, given as a discrete number and unit of measurement
☐	☒	A statement on whether measurements were taken from distinct samples or whether the same sample was measured repeatedly
☐	☒	The statistical test(s) used AND whether they are one- or two-sided<br>*Only common tests should be described solely by name; describe more complex techniques in the Methods section.*
☐	☒	A description of all covariates tested
☐	☒	A description of any assumptions or corrections, such as tests of normality and adjustment for multiple comparisons
☐	☒	A full description of the statistical parameters including central tendency (e.g. means) or other basic estimates (e.g. regression coefficient) AND variation (e.g. standard deviation) or associated estimates of uncertainty (e.g. confidence intervals)
☐	☒	For null hypothesis testing, the test statistic (e.g. *F*, *t*, *r*) with confidence intervals, effect sizes, degrees of freedom and *P* value noted<br>*Give P values as exact values whenever suitable.*
☒	☐	For Bayesian analysis, information on the choice of priors and Markov chain Monte Carlo settings
☒	☐	For hierarchical and complex designs, identification of the appropriate level for tests and full reporting of outcomes
☒	☐	Estimates of effect sizes (e.g. Cohen's *d*, Pearson's *r*), indicating how they were calculated

Our web collection on statistics for biologists contains articles on many of the points above.

Software and code

Policy information about availability of computer code

Data collection	This information is described in the 'Materials and Methods' section.
Data analysis	This information is described in the figure legends and 'Materials and Methods' section.

For manuscripts utilizing custom algorithms or software that are central to the research but not yet described in published literature, software must be made available to editors and reviewers. We strongly encourage code deposition in a community repository (e.g. GitHub). See the Nature Portfolio guidelines for submitting code & software for further information.

Data

Policy information about availability of data

All manuscripts must include a data availability statement. This statement should provide the following information, where applicable:

- Accession codes, unique identifiers, or web links for publicly available datasets
- A description of any restrictions on data availability
- For clinical datasets or third party data, please ensure that the statement adheres to our policy

All the data generated or analyzed in this study are included in the figures, texts, and supplementary information files. . The bulk RNA seq data of OPCs and neurons generated in this study have been deposited in the GEO repository database under accession code GSE285995. Source data are provided with this paper.

Research involving human participants, their data, or biological material

Policy information about studies with human participants or human data. See also policy information about sex, gender (identity/presentation), and sexual orientation and race, ethnicity and racism.

Reporting on sex and gender	N/A
Reporting on race, ethnicity, or other socially relevant groupings	N/A
Population characteristics	N/A
Recruitment	N/A
Ethics oversight	N/A

Note that full information on the approval of the study protocol must also be provided in the manuscript.

Field-specific reporting

Please select the one below that is the best fit for your research. If you are not sure, read the appropriate sections before making your selection.

☒ Life sciences ☐ Behavioural & social sciences ☐ Ecological, evolutionary & environmental sciences

For a reference copy of the document with all sections, see nature.com/documents/nr-reporting-summary-flat.pdf

Life sciences study design

All studies must disclose on these points even when the disclosure is negative.

Sample size	Samples sizes are described in figure legends. No statistical methods were used to pre-determine sample sizes. In all animal experiments, 3-10 mice (indicated in figure legends) were analyzed per group. For in vitro studies, experiments are performed 3-5 times independently (indicated in figure legends).
Data exclusions	No data were excluded.
Replication	For in vivo experiments, littermates from different breeding pairs were studied and the data were pooled. (Materials and Methods).
Randomization	Littermates with different genotypes were assigned to corresponding groups, i.e. ctl and cKO groups.
Blinding	Yes, the studies for image analysis and cell culture experiments were performed blind. (Materials and Methods).

Reporting for specific materials, systems and methods

We require information from authors about some types of materials, experimental systems and methods used in many studies. Here, indicate whether each material, system or method listed is relevant to your study. If you are not sure if a list item applies to your research, read the appropriate section before selecting a response.

Materials & experimental systems

n/a	Involved in the study	
☐	☒	Antibodies
☒	☐	Eukaryotic cell lines
☒	☐	Palaeontology and archaeology
☐	☒	Animals and other organisms
☒	☐	Clinical data
☒	☐	Dual use research of concern
☒	☐	Plants

Methods

n/a	Involved in the study	
☒	☐	ChIP-seq
☒	☐	Flow cytometry
☒	☐	MRI-based neuroimaging

Antibodies

Antibodies used	Yes, described in detail in the supplementary tables 1-2
Validation	Each antibody has been widely used and reported. Relevant information is available on the vendor's website.

Animals and other research organisms

Policy information about studies involving animals; ARRIVE guidelines recommended for reporting animal research, and Sex and Gender in Research

Laboratory animals	Details of animals used in the study are listed in the 'Animals' section of the 'Materials and Methods'.
Wild animals	None
Reporting on sex	We have pooled the data from both the males and females, as no sex-dependent difference has been observed.
Field-collected samples	None
Ethics oversight	Majority of the experiments were carried out at the University of Saarland in strict accordance with recommendations of European and German guidelines for the welfare of experimental animals. Animal experiments were approved by Saarland state's "Landesamt für Gesundheit und Verbraucherschutz" in Saarbrücken/Germany (animal license numbers: 65/2013, 12/2014, 34/2016, 36/2016, 03/2021, 07/2021, 08/2021, Perfusion-2023, VM2024-03 and 17/2023) and 'Laboratory animal welfare and ethics committee' of the Third Military Medical University, China (SYXK (YU) 2022-0018).

Note that full information on the approval of the study protocol must also be provided in the manuscript.

Plants

Seed stocks	N/A
Novel plant genotypes	N/A
Authentication	N/A
